# Novel transcripts of EMT driving the malignant transformation of oral submucous fibrosis

**DOI:** 10.1038/s41598-025-87790-2

**Published:** 2025-01-26

**Authors:** Smitha Sammith Shetty, Kanaka Sai Ram Padam, Mohit Sharma, Adarsh Kudva, Pratik Patel, Raghu Radhakrishnan

**Affiliations:** 1 Department of Oral and Maxillofacial Pathology and Microbiology, Manipal College of Dental Sciences, Manipal Academy of Higher Education, Manipal, 576104 India; 2 Department of Cell and Molecular Biology, Manipal School of Life Sciences, Manipal Academy of Higher Education, Manipal, Karnataka India; 3 Department of Oral Pathology, SGT Dental College Hospital & Research Institute, Gurugram, 122505 Haryana India; 4 Department of Oral and Maxillofacial Surgery, Manipal College of Dental Sciences, Manipal Academy of Higher Education, Manipal, 576104 India; 5 Sangee Oral Pathology Center, Haripura, Surat, 395003 Gujarat India; 6 Unit of Oral and Maxillofacial Pathology, School of Clinical Dentistry, University of Sheffield, Sheffield, S102TA UK; 7 Unit of Oral and Maxillofacial Pathology, Oman Dental College, P.O Box 835, Muscat, Wattayah 116 Oman

**Keywords:** Oral squamous cell carcinoma, Oral submucous fibrosis, Candidate genes, EMT, *In-silico* analysis, Proteomics, Cancer, Oncology

## Abstract

Oral submucous fibrosis (OSF) is a chronic, progressive, and fibrotic condition of the oral mucosa that carries an elevated risk of malignant transformation. We aimed to identify and validate novel genes associated with the regulation of epithelial-to-mesenchymal transition (EMT) in OSF. Genes regulating EMT were identified through differential gene expression analysis, using a LogFC threshold of -1 and + 1 and a padj value < 0.05, based on data from GEO datasets and the TCGA-HNSC datasets. The curated EMT genes were correlated with functional cancer states and subjected to clustering to identify candidate genes. Integration of bioinformatics and proteomics led to the discovery of the EMT genes *MMP9*, *SPARC*, and *ITGA5* as novel candidates. Comprehensive pathway and immunohistochemical analyses confirmed their roles in regulating EMT in OSF, oral squamous cell carcinoma (OSCC), and OSF-associated squamous cell carcinoma (OSFSCC). The significant roles of *MMP9*, *SPARC*, and *ITGA5* in fibrosis and malignancy suggest a novel mechanism in which fibrosis-associated type 2 EMT undergoes transition to type 3 EMT, driving OSF towards malignancy.

**Supplementary Information:**

The online version contains supplementary material available at 10.1038/s41598-025-87790-2.

## Introduction

Oral cancer, encompassing lip, mouth, and oropharynx cancers, ranks as the 13th most common cancer globally. In 2020, an estimated 377,713 new cases and 177,757 deaths occurred globally from lip and oral cavity cancers^1^. Most of oral cancer progression can be traced from precursor lesions, referred to as oral potentially malignant disorders (OPMDs). Various retrospective and epidemiological studies have shown the progression of oral leukoplakia, oral lichen planus (OLP), and oral submucous fibrosis (OSF) to oral squamous cell carcinoma (OSCC)^2^. A number of cases of OSCC have been documented as originating from OSF as a result of the widespread consumption of betel quid^3^. The malignant transformation rate of OSF is high, ranging from 7 to 13%, and varies by race, area, lifestyle, and culture^4^. Recently, meta-analyses and systematic reviews have shown that the global proportions of OSF patients who undergo malignant transformation are 5.2% and 4.6%, respectively^5,6^. Oral cancer arising from OSF is a distinct entity due to varied clinicopathological, morphological and histological features attributed to areca nut-induced carcinogenesis. Additionally, as OSF is a connective tissue disorder, the epithelial alterations observed during malignant transformation require further explanation as to whether the changes are induced by stromal fibrosis or the cumulative impact of areca nuts on the epithelium. If it is the areca nut that induces carcinomatous changes, then why do not all cases transform into malignancies?

OSF is characterized by changes in squamous epithelium ranging from atrophy to hyperplasia and/or dysplastic changes with fibrotic alterations in the connective tissue stroma^7^. These alterations could drive the malignant transformation of OSF patients associated with unaccounted risk habits and genomic alterations in key cancer-associated genes^7^. The epithelial cells in such a milieu demonstrate loss of cellular adhesion and polarity, exhibiting epithelial mesenchymal transition (EMT).

The process by which epithelial cells lose polarity and cell‒cell adhesion to acquire the properties of mesenchymal cells is known as epithelial–mesenchymal transition (EMT)^8^. The evidence in the literature on the upregulation of EMT-specific markers in OSF and OSCC suggests that EMT-mediated molecular networks participate in the development and progression of fibrosis and cancer^8–11^. The intriguing link between EMT in OSCC^12^ and OSF progression to malignancy^13^ prompts the exploration of candidate genes regulating EMT changes.

High-throughput, genome-wide studies, such as gene expression profiling, which examine the entire genome, prove valuable for categorizing and characterizing disease conditions. To prioritize genes, various computational techniques using publicly available datasets can be integrated with genome-wide studies to identify potential candidate genes^14^. In this study, we explored available data mining tools, examined the biological sequences in our clinical samples, performed expression and phenotype analyses, investigated protein‒protein interactions (PPIs), and constructed gene regulatory networks and pathways linked to the promotion of EMT in OSF and the initiation of malignant transformation^15,16^.

## Results

### EMT-regulating genes

A total of 1185 profiles of EMT genes were accessed from dbEMT, and data from 85 research papers were downloaded from EMTome. The combined list of EMT signatures reported across cancer types included approximately 4812 genes. The expression of these curated EMT signature genes was analysed in the 2 datasets retrieved from the GEO dataset and TCGA-Oral Cavity subset.

### Data retrieved from databases

A study with the GEO accession number GSE64216^17^ was considered, and data relevant to NOM, OSF and OSFCC (OSF associated with OSCC) were retrieved. A dataset specific to the oral cavity subset consisting of 18 normal and 234 primary tumors was retrieved from the TCGA^18^.

### Differentially expressed genes (DEGs)

Differential gene expression analysis of the Geo dataset (GSE64216) revealed distinctive patterns among the subgroups. A total of 209 DEGs, consisting of 86 upregulated and 123 downregulated genes, were found in the matched OSFWT vs. NOM. In the matched OSFSCC vs. NOM cohort, 153 DEGs were found, with 69 upregulated and 84 downregulated genes. Analysis of the TCGA-HNSC-OC database for NOM vs. OSCC revealed a total of 288 DEGs, with 170 upregulated and 118 downregulated genes.

### Positive correlation of the curated EMT gene list with the functional states of cancers

The DEGs identified from the analysis of two datasets (TCGA-HNSC, GEO dataset, and whole transcriptomic data) using the pathway activity module of GSCALite showed activation or inhibition of various pathways related to apoptosis, the cell cycle, DNA damage response, hormone ER, hormone AR, EMT, PI3K/AKT, RAS/MAPK, RTK etc., across 32 different cancers. The top 10 genes activating the EMT pathway were shortlisted from the NOM vs. OSFWT, NOM vs. OSCC and NOM vs. OSFSCC datasets, and the final top 30 genes were selected based on the percentage of cancers in which these genes activate EMT pathway. The heatmap shows the consolidated list of the top genes implicated in the activation of EMT across various cancers (Supplementary Fig. 1)

**Fig. 1:**
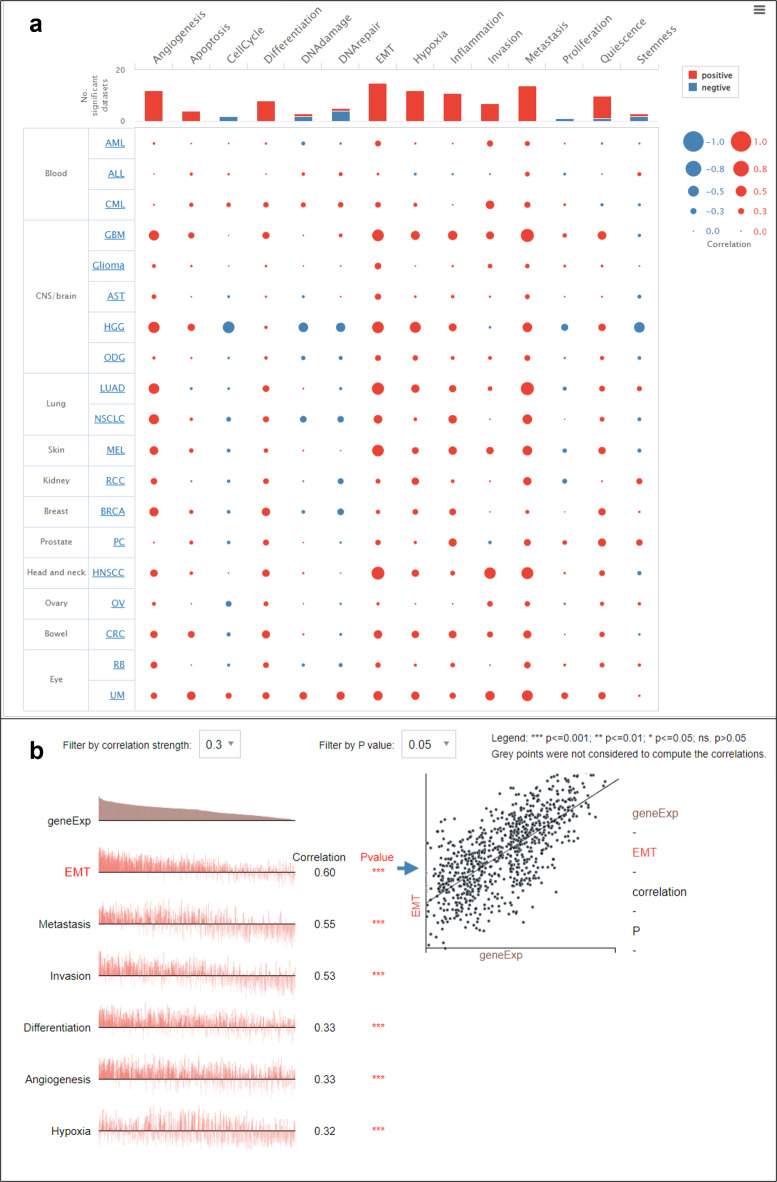
The correlation plot shows the positive correlation between the curated EMT gene list and functional states in various cancers, such as HNSCC, skin (melanoma), lung (lung adenocarcinoma), and brain (high-grade glioma, glioblastoma) (**a**). Scatter plots showing the positive correlation between curated gene expression and EMT and other functional states of cancer (**b**).

On correlating the expression of the curated list of genes with the hallmarks of cancer, such as angiogenesis, apoptosis, cell cycle, EMT, hypoxia, DNA damage, invasion, and metastasis stemness, we found a positive correlation between curated gene expression and EMT in HNSCC, melanoma, lung adenocarcinoma, and high-grade glioma (Fig. 1a).

The correlation between the curated gene expression and functional states of cancer in HNSCC showed a statistically significant (*p* > 0.05) positive association with functional states such as EMT, metastasis, invasion, differentiation, angiogenesis and hypoxia in HNSCC (Fig. 1b)

### Enriched functional GO terms and pathways

GO and pathway enrichment analyses of the curated genes revealed 109 enriched GO terms (81 BP, 8 MF and 20 CC) (FDR < 0.05). However, given the considerable number of genes, many genes that are predicted may be false positives. To improve efficiency, the GO terms that exhibited associations with a gene count less than 3 were subjected to filtration to eliminate nonspecific terms^19^. Filtration with this method yielded a total of 103 GO terms (79BP, 7MF and 17 CC) (FDR < 0.05). The highly enriched GO terms (BP) included regulation of cell adhesion, cell adhesion, regulation of cell migration, cell migration, regulation of cell motility, etc. (Supplementary Fig. 2a). Similarly, the enriched GO terms (MFs) included extracellular matrix structural constituent, collagen binding, signalling receptor binding, cell adhesion molecule binding, etc. (Supplementary Fig. 2b). The enriched GO terms (CCs) included extracellular matrix, collagen-containing extracellular matrix, protein complex involved in cell adhesion, etc. (Supplementary Fig. 2c). All the enriched terms were strongly associated with EMT.

The curated EMT genes subjected to pathway analysis revealed 19 pathways that were significantly enriched (*p* > 0.05). Among the highly enriched pathways in the WikiPathways category were EMT in colorectal cancer and senescence, autophagy in cancer, and the TGFβ/Smad signalling pathway. In the KEGG category, the highly enriched pathways was proteoglycans and cancer^20^, while in the Reactome category, the highly enriched pathways included extracellular matrix organization and integrin cell surface interactions (Supplementary Fig. 3). The enriched pathways also implicate the involvement of the curated genes in EMT.

### Novel candidate genes

**Fig. 2:**
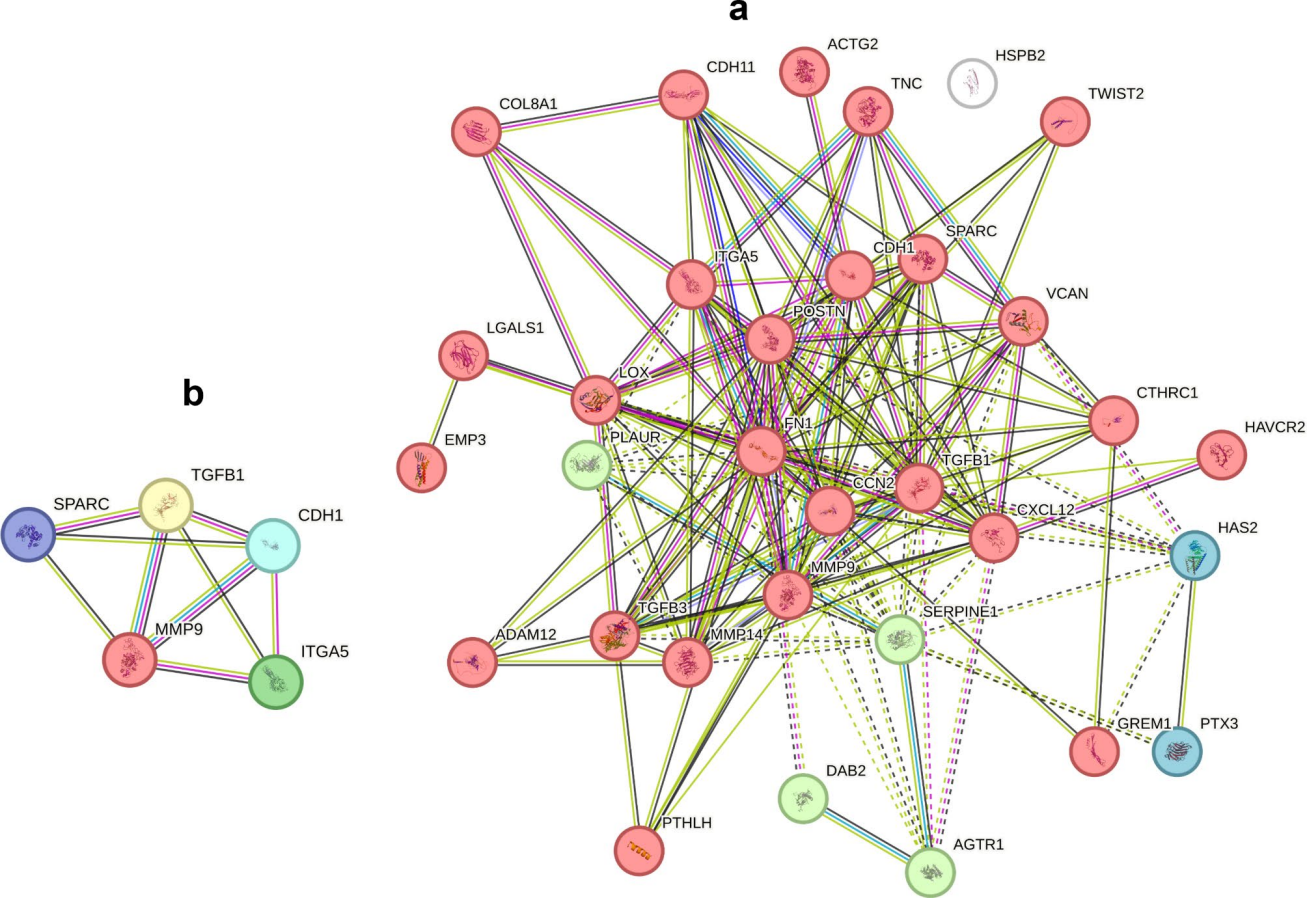
Cluster analysis of curated EMT genes using STRINGbd. The three clusters that were identified are visually represented by the colours red, green, and blue (**a**). Protein‒protein interactions (PPIs) were determined through the use of the STRING database to visualize the interactions involving the candidate genes MMP9, SPARC and ITGA5 (**b**).

A cluster analysis of the curated genes identified 3 clusters consisting of 32 genes distributed across three distinct clusters, denoted as clusters A, B, and C. Of the three clusters, cluster A (red) comprised the greatest number, 25 genes, while cluster B (green) contained a total of 4 genes. In contrast, cluster C (blue) displayed a notably small number of genes, consisting of only 2 genes. Based on the maximum PPI with nodes of *TGF-β* and *CDH1*, the following candidate genes, MMP9, SPARC and ITGA5, were predicted to regulate EMT (Fig. 2).

### Upregulation of the novel candidate genes MMP9, SPARC and ITGA5

**Fig. 3:**
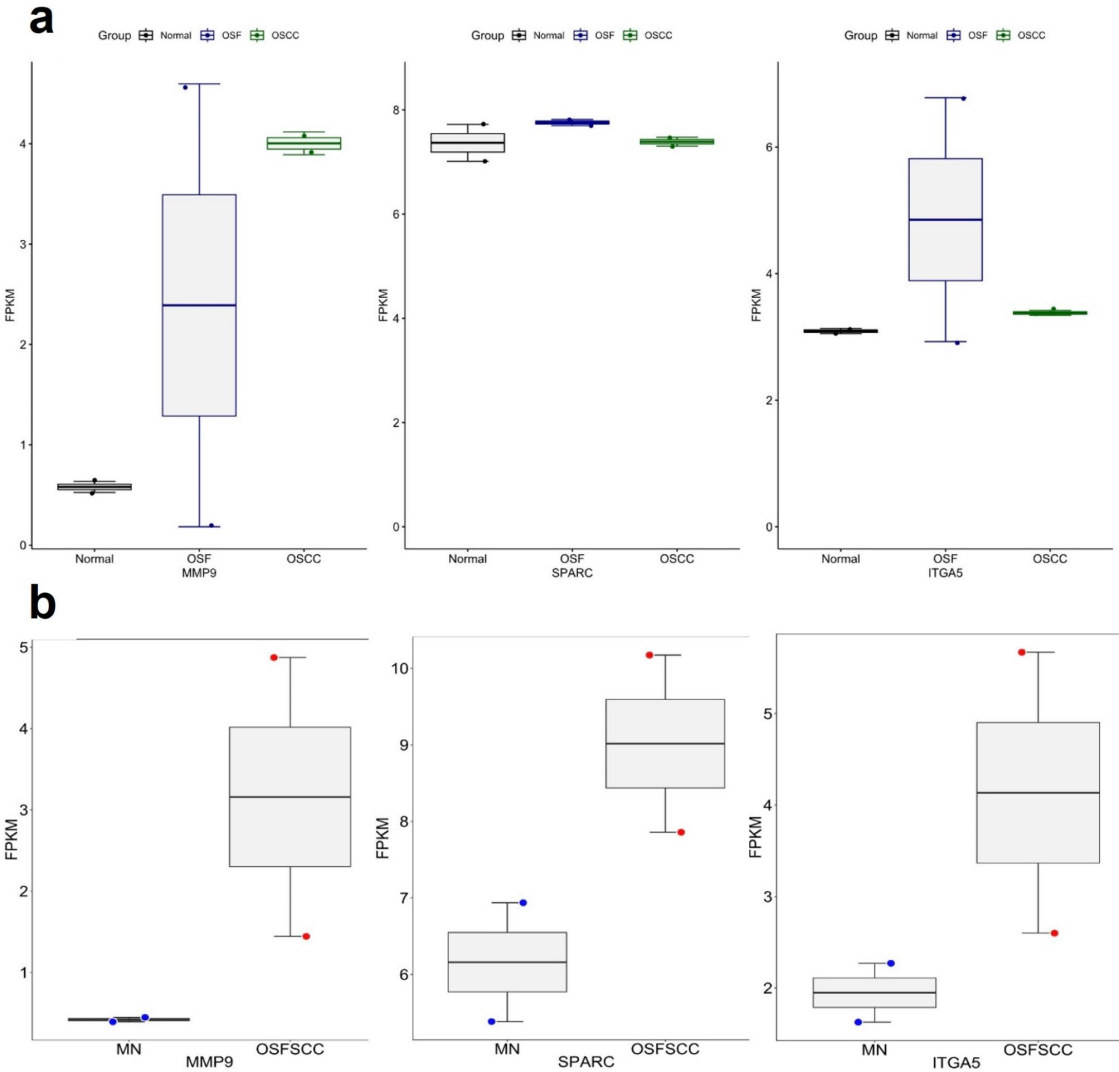
Distribution of FPKM data for MMP9, SPARC and ITGA5 derived from whole-transcriptome analysis between the NOM, OSF, OSFSCC, and OSCC groups (**a**). mRNA transcriptome profiles of MMP9, SPARC and ITGA5 in OSFSCC tissues compared with those in matched normal (MH) tissues (**b**).

Analysis of the expression profiles of the candidate genes via RNA sequencing revealed that MMP9 was upregulated in OSCC samples compared to OSF and NOM samples. SPARC was more highly expressed in OSF and OSCC samples than in NOM samples. ITGA5 was also more highly expressed in OSF and OSCC patients than in NOM patients. The expression of MMP9, SPARC and ITGA5 was significantly upregulated in OSFSCC patients compared to matched NOM patients (Fig. 3).

### Validation by immunohistochemistry

**Fig. 4:**
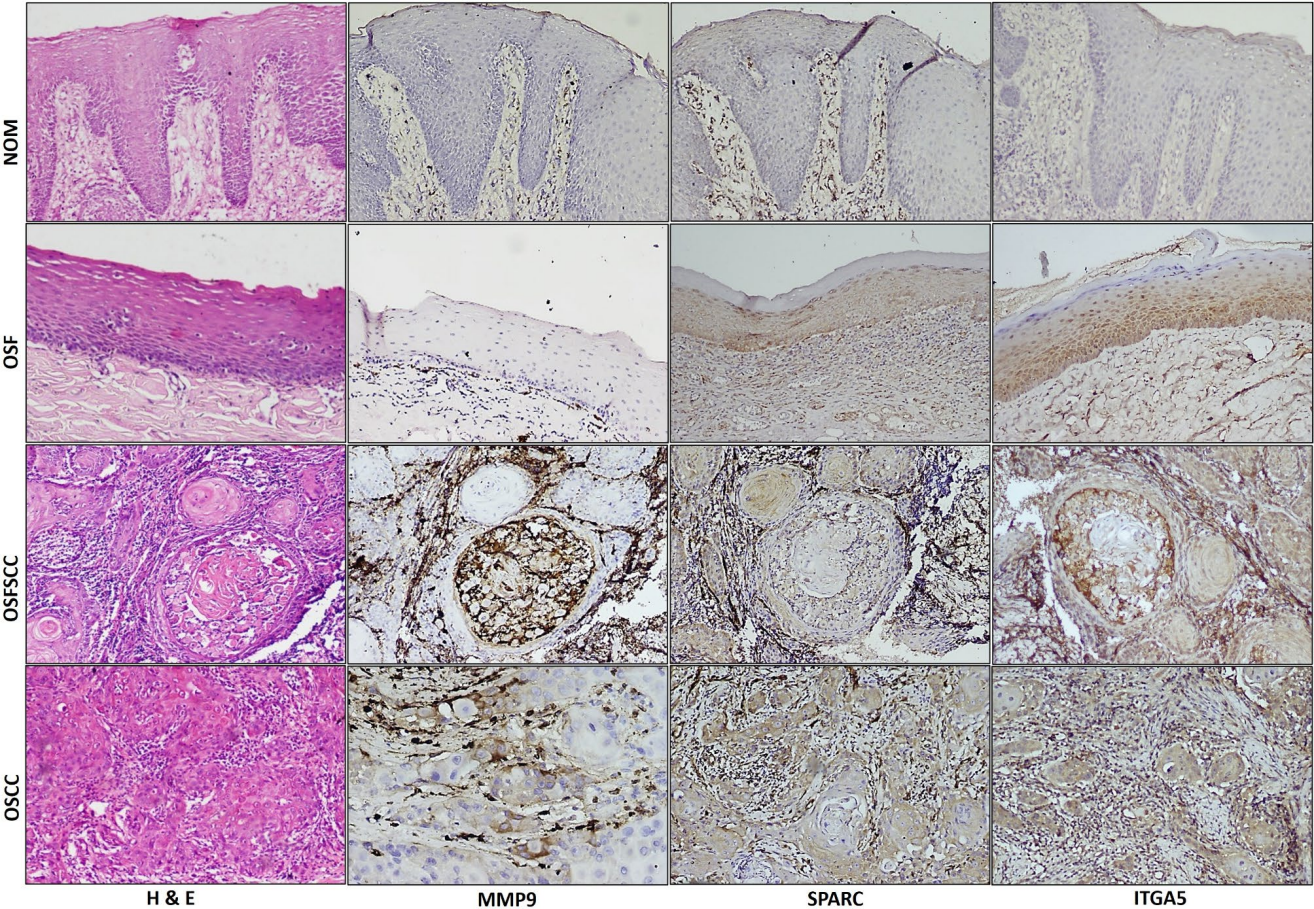
Immunohistochemical analysis of candidate genes in NOM, OSF, OSFSCC and OSCC. SPARC and ITGA5 exhibited cytoplasmic staining in OSF extending up to the spinous region and in the peripheral cells of the tumour islands in oral squamous cell carcinomas associated with OSF (OSFSCC) and oral squamous cell carcinoma (OSCC). MMP9 lacked staining in OSFs and was cytoplasmic in tumour cells in the area of invasion and in tumour islands.

**Fig. 5:**
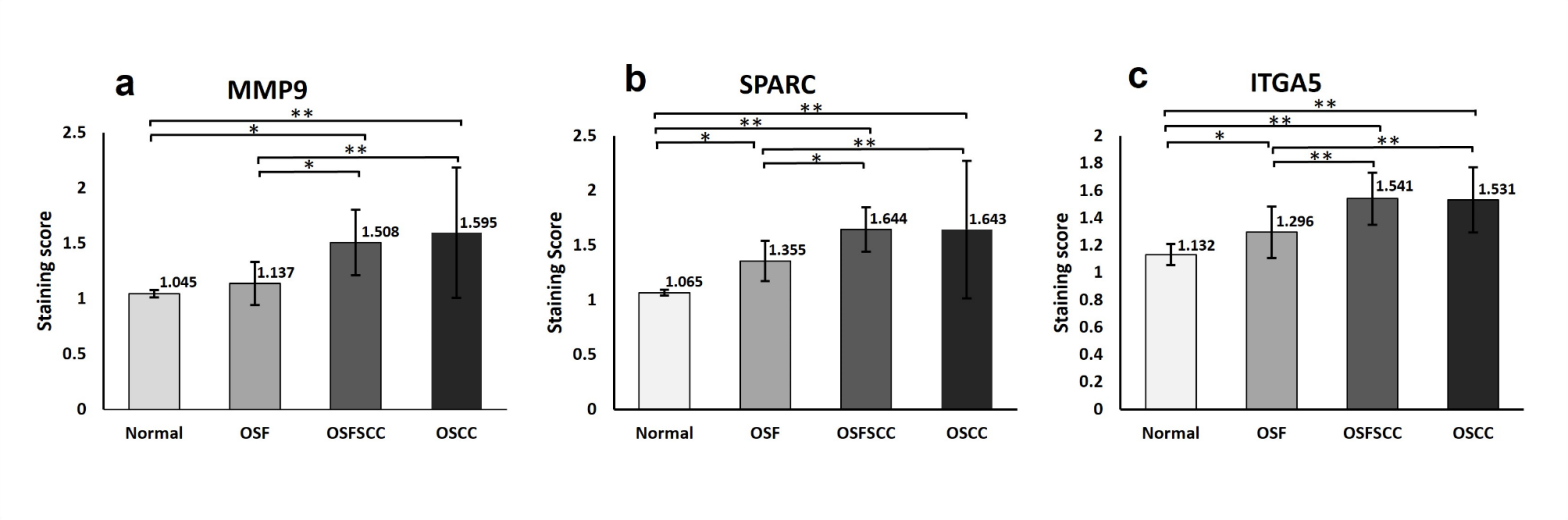
Comparison of MMP9 (**a**), SPARC (**b**) and ITGA5 (**c**) expression among the NOM, OSF, OSFSCC, and OSCC groups. Each bar represents the mean ± SD, *P < 0.05, **P < 0.001.

Immunohistochemical analysis revealed the localization of MMP9, SPARC and ITGA5 predominantly within the cytoplasmic compartment of the neoplastic cells (Fig. 4). The immunoreactivity of SPARC and ITGA5 was significantly greater in OSFSCC (*p* < 0.001), OSCC (*p* < 0.001) and OSF (*p* < 0.05) than in NOM. MMP9 was significantly more highly expressed in OSFSCC (*p* < 0.001) and OSCC (*p* < 0.001) patients than in OSF and NOM patients. Nevertheless, the expression levels of the candidate genes did not significantly differ between OSFSCC and OSCC, as illustrated in Fig. 5.

## Discussion

EMT is a biological phenomenon wherein an epithelial cell undergoes a transformative process, adopting a mesenchymal phenotype. Based on the biological context of its occurrence, it is classified as Type 1, which occurs during embryogenesis; Type 2, which is associated with wound healing and fibrosis; and Type 3, which is involved in tumor invasion, progression and metastasis^21^. In cancer biology, type 3 is of interest because it plays a crucial role in facilitating tumour migration, conferring cancer stem cell characteristics, promoting resistance to chemotherapy, and enhancing metastatic capabilities^22^. Intriguingly, OSF, a fibrotic disorder, has significant potential to undergo malignant changes. This raises the question of whether type 2 EMT plays a crucial role in the progression of fibrosis in OSF and thereby creates a microenvironment conducive to facilitating its transition to type 3 EMT. While the role of EMT has been highlighted in OSCC^12^, it is essential to understand how EMT contributes to the progression of OSF to OSCC^13^.

DEGs derived from the datasets showed a positive correlation with the functional states of cancer, such as EMT, differentiation, angiogenesis, invasion, hypoxia, and metastasis, across various cancers. Pathway analysis also revealed the relevance of EMT in cancer.

Following in silico predictions, the candidate genes MMP9, SPARC and ITGA5 were validated through transcriptome profiling and immunohistochemistry. Matrix metalloproteinases (MMPs) are a group of zinc-dependent endopeptidases that can breakdown different constituents of the extracellular matrix (ECM). In this family, MMP9 plays a significant role in ECM remodelling by degrading collagen and other ECM components. The overexpression of MMP9 contributes to the excessive degradation of the ECM, altering the tissue microenvironment to induce inflammatory responses, activating fibroblasts to promote fibrosis^23^. MMP9 also cleaves the latency-associated peptide (LAP) bound to latent TGF-β, resulting in the release of active TGF-β to promote the pro-fibrotic pathway^24^.

In addition, MMP-9, referred to as gelatinase-B or type IV collagenases serves as a crucial constituent of the basement membrane^25^. MMP-9 primarily facilitates the degradation of Collagen IV, which is the principal constituent of the basement membrane (BM) and extracellular matrix (ECM)^25^. MMP-2 and MMP-9 have been extensively investigated in the context of invasion, primarily due to their pivotal role in the degradation of the basement membrane^26^.

Our study revealed that MMP-9 was overexpressed at both the transcriptional and translational levels in OSFSCC and OSCC patients compared to OSF and NOM patients. The differential expression of MMP-9 in the transcriptome data indicated the role of MMP-9 in the pathogenesis of OSF, OSFSCC and OSSCC. Elevated expression of MMP-9 has been reported in precancerous lesions as well as in oral cancer^27^. The upregulation of MMP-9 in OSCC cells indicates a potential correlation with proliferation, invasion, and metastasis^28^.

**Fig. 6:**
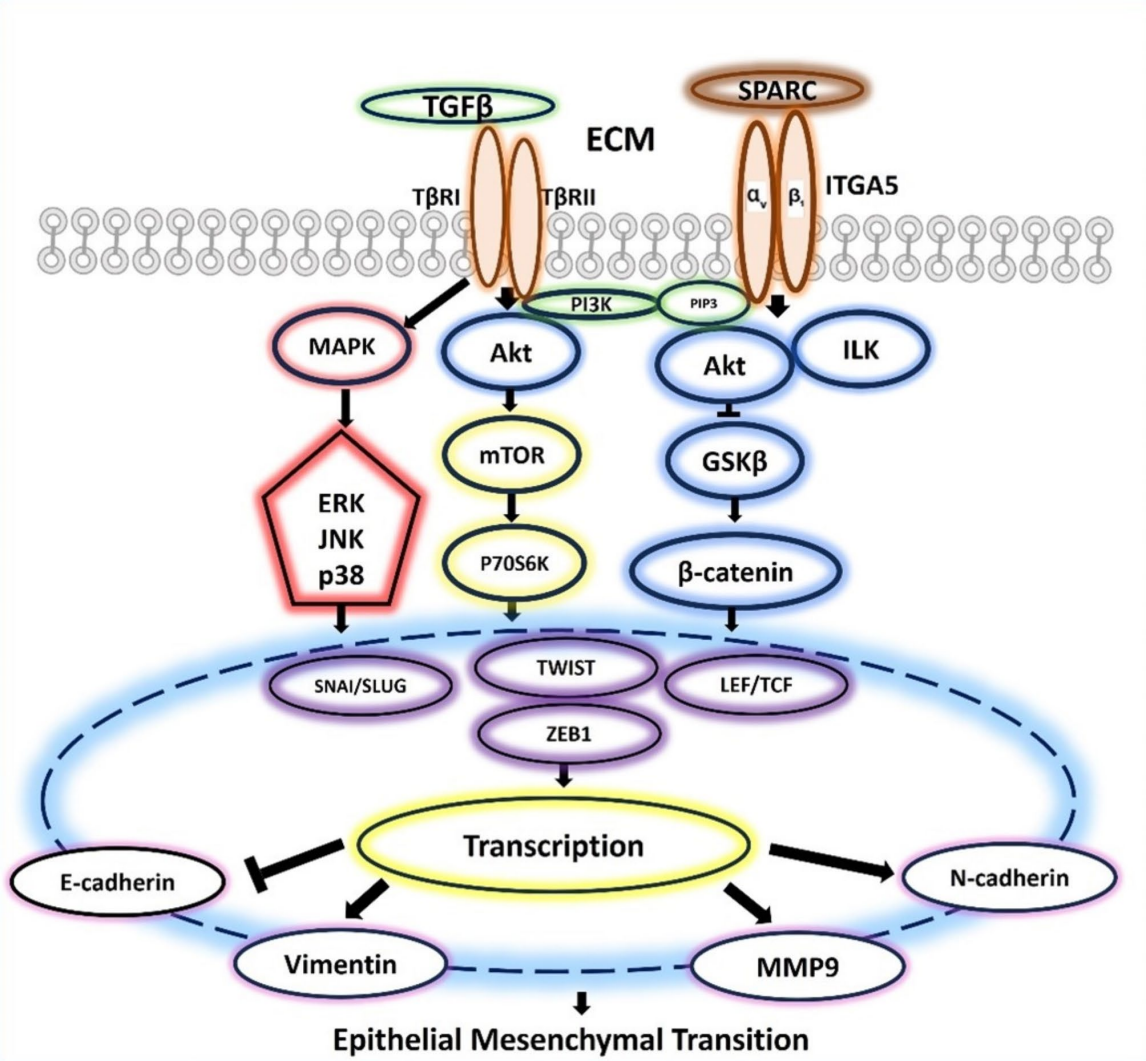
Schematic illustration of the proposed SPARC-, MMP9- and ITGA5-mediated signalling pathways that induce EMT.

It has been well documented that the initiation of the EMT mechanism in OSF is associated with elevated expression of MMP-9^8,29^. Moreover, arecoline can stimulate keratinocytes, leading to the secretion of MMP-9, thereby promoting microinvasion and triggering the onset of oral carcinogenesis^27^. The arecoline-induced upregulation of MMP-9 is mediated via the p38 MAPK, STAT3 and NF-κB/IκB pathways^27^ (Fig. 6). MMP-9 functions as a downstream target of TGF-β1, and the repression of E-cadherin induced by TGF-β1 is primarily facilitated by MMP-9, thus promoting the process of EMT^30^. Network analysis confirmed that MMP9 is a potential candidate involved in oral carcinogenesis and could thus serve as a potential drug target^31^.

SPARC, referred to as osteonectin or basement membrane-40 (BM-40), belongs to a group of matricellular proteins that regulate the interactions between cells and the extracellular matrix^32^. Additionally, SPARC interacts with cell surface receptors and various growth factors to modulate a wide range of biological processes associated with physiological homeostasis and pathological conditions^32^. Although evidence supports the significance of SPARC in various cancer types, a comprehensive understanding of its multifaceted function and impact on cancer development and progression remains elusive^33^.

The expression of SPARC in OSF has not been investigated. However, previous in vitro and in vivo studies on fibrosis have revealed the role of SPARC in regulating collagen expression by influencing the expression of CTGF, which is a biomarker for TGF-β activity. A positive feedback loop exists between SPARC and TGF-β and is mediated via CTGF to induce apoptosis and fibrosis^32^. Hence, silencing SPARC can attenuate TGFβ-induced fibrosis^34^. In silico analysis predicted that SPARC is enriched and a significant marker regulating EMT in OSF^35^, and it is also differentially expressed in OSCC^36^. Furthermore, SPARC contributes to tumour progression by inducing EMT via the activation of SNAI2^37^. In our cohort, SPARC exhibited upregulated expression at the protein and RNA levels in OSF, OSFSCC, and OSCC compared to NOM. The upregulation of SPARC in OSCC suggests its involvement in the transformative alterations of the epithelium as well as its potential as an early event in the process of cancer development^38^. SPARC facilitates the promotion of proliferation and metastasis in OSCC through the activation of EMT pathways, such as the PI3K/AKT/PDGFB/PDGFRβ axis^38^ or the PI3K/AKT and MAPK signalling pathways^37^ (Fig. 6). These findings indicate that SPARC acts as a positive regulator of both fibrosis^32^ and malignancy^39^ through EMT. This contributes to the cyclic progression from fibrosis to malignancy in OSF, initiating a transition from type 2 EMT to type 3 EMT.

Integrins are a diverse group of glycoprotein transmembrane receptors that play crucial roles in facilitating cell-to-extracellular matrix and cell-to-cell adhesion. Integrins are composed of heterodimers comprising both α and β subunits^40^. Integrin subunit α5 (ITGA5) frequently associates with integrin β1 (ITGB1) to form the heterodimer Integrin α5β1, which functions as a receptor facilitating cellular differentiation, development, and migration^41^. ITGA5 plays a crucial role in mediating the communication between the ECM and stromal cells; hence, its upregulation is associated with increased proliferation of fibroblasts leading to fibrosis^42^. Further growing research has substantiated the enhanced expression of ITGA5 in various malignancies, establishing its association with tumour advancement^40^.

Increased expression of ITGA5 at both the RNA and protein levels was evident in OSF, OSFSCC and OSCC patients in our cohort. While the overexpression of ITGA5 has been associated with systemic sclerosis^43^ and idiopathic pulmonary fibrosis^42^, our study is the first to attempt to understand the functional significance of ITGA5 in the pathogenesis of OSF. The upregulation of ITGA5 is functionally linked to myofibroblast differentiation through TGF-β through a feed forward loop^42^. This highlights the interplay of integrin and TGF-β in fibrosis. ITGA5 serves as the primary receptor for fibronectin (FN); hence, the binding of the two proteins is essential for cell adhesion and migration. Upon upregulation, there may be increased interaction between ITGA5 and FN, leading to activation of the profibrotic PI3K/AKT pathway and resulting in fibrosis^44^. ITGA5 can function as an oncogene, promoting proliferation, migration and invasion by activating EMT in carcinomas^41^. Biologically silenced ITGA5 inhibits the proliferation, migration and invasion of OSCC cells^45^. The cancer cells exhibiting an upregulation of ITGA5 demonstrated EMT-associated markers, characterized by the repression of epithelial markers and the overexpression of mesenchymal markers^41^. The upregulation of ITGA5, which is downstream of the PI3K/AKT signalling pathway, is positively correlated with the expression of PI3K and AKT, indicating that ITGA5 plays a significant role in OSCC progression^45^ (Fig. 6). Increased expression of ITGA5, at both the transcriptional and translational levels, provides substantial evidence of its involvement in fibrosis and malignancy through the induction of EMT.

The upregulation of SPARC, MMP9 and ITGA5 in OSF and OSCC indicates their role in the induction of fibrosis and progression to cancer. SPARC, an upstream regulator of CTGF in response to TGFβ stimulation, modulates the ECM by regulating collagen production and assembly in various fibrotic diseases^34^. SPARC cooperates with TGFβ signalling for pro-fibrotic activation, inducing fibrosis^32^. SPARC can also induce EMT by upregulating the expression of EMT regulatory transcription factors SNAI1/2 and ZEB1 thereby inducing changes in cellular phenotype to promote tumour progression^46^. While SPARC can induce MMP9 secretion from the fibroblasts^47,48^, MMP9 can further facilitate the release of active pro-fibrotic TGF-β1 through proteolytic cleavage of LAP bound to TGF-β1 promoting fibrosis^24^. Thus, positive feedback exists between SPARC and MMP9.

**Fig. 7:**
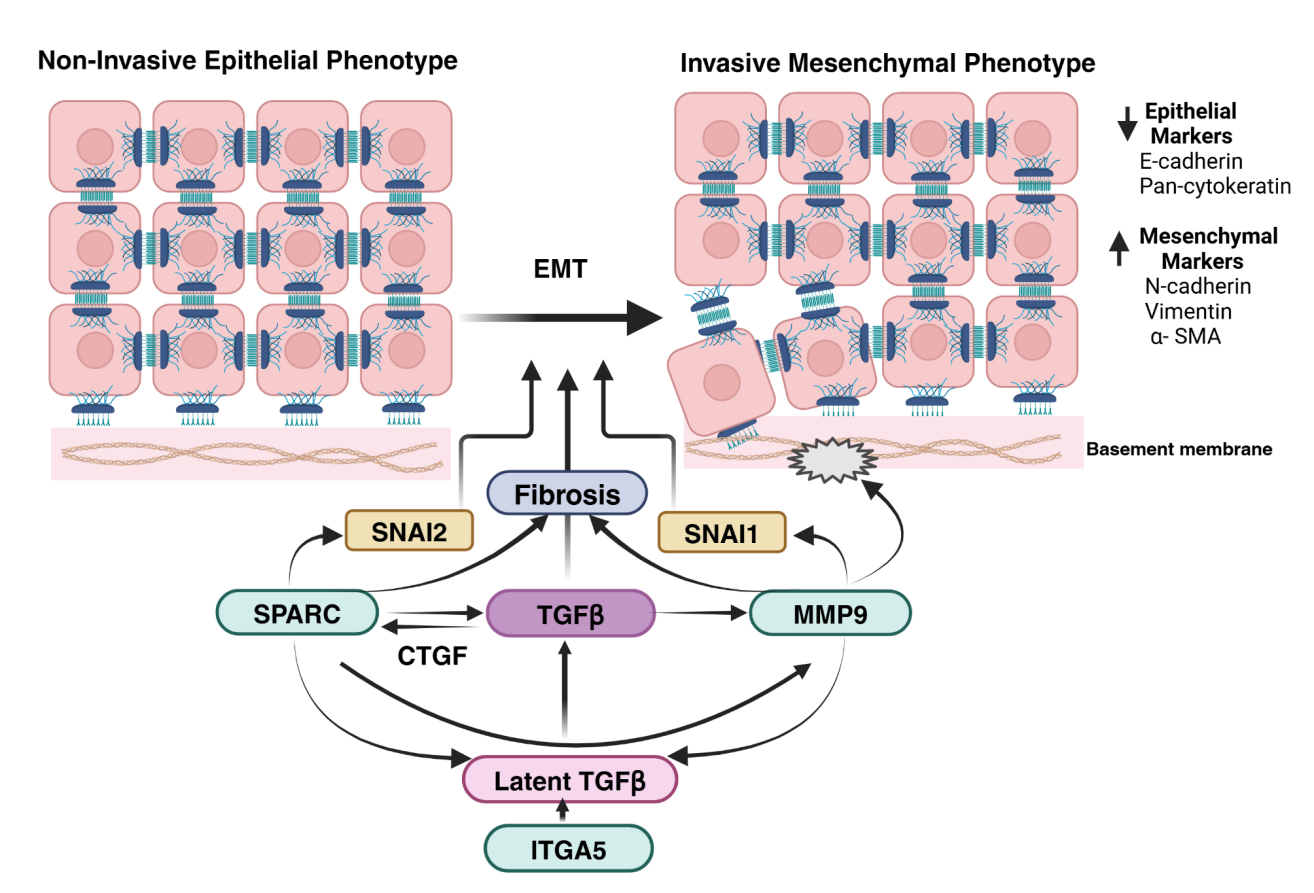
The proposed crosstalk between SPARC, MMP9 and ITGA5 in inducing EMT.

MMP9 induces ECM and basement membrane degradation, facilitating microinvasion, proliferation and migration of tumour cells to promote EMT^27,49^. TGF-β1 promotes MMP9 mediated oral cancer invasion by upregulating the transcription factor SNAI1^50^. ITGA5, a receptor of fibronectin can be upregulated by CTGF and TGFβ contributing to fibrosis^51^. ITGA5 also functions as the transmembrane receptor, facilitating SPARC-induced ECM changes mediated through fibronectin, which activates fibroblasts to induce dominant ECM alterations that promote tumour cell proliferation and migration in the stroma^52,53^. ITGA5 enhances the cell proliferative, migrative and invasive abilities by EMT to promote the malignant advancement of OSCC^41,54^ (Fig. 7). Thus, the literature and our results support the hypothesis that these molecules contribute to ECM deposition, fibroblast activation, and fibrosis, which characterize OSF. The progression of OSF toward malignancy is associated with the upregulation of SPARC, activation of MMP9, and overexpression of ITGA5, promoting ECM degradation, facilitating cell invasion, and driving tumor progression. This indicates a switch from fibrosis-associated type 2 EMT to type 3 EMT, which is involved in malignant transformation.

The therapeutic potential of targeting MMP9, SPARC, and ITGA5 is heightened when considered in combination, as they are involved in different but complementary pathways of ECM remodelling, fibrosis, and tumor progression. Thus, targeting these molecules may help prevent the progression of fibrosis and inhibit the malignant transformation of OSF into OSCC. The inhibition of MMP9 not only prevents ECM degradation but can also inhibit the activation of myofibroblast, potentially reversing or halting the progression of fibrosis^23^ in OSF patients. Additionally, inhibiting MMP9 suppresses ECM degradation and thereby reduces the invasive properties of cancer cells^55^ possibly preventing the malignant transformation of OSF into OSCC. Similarly, SPARC and ITGA5 are potential targets to reduce ECM deposition by preventing the activation of myofibroblasts and inhibiting fibrosis^56,57^. Moreover, they may impede tumour progression by blocking the cell-ECM interactions essential for cancer cell proliferation, migration and invasion^38,58^.

Research on potential therapeutic implications has been conducted on several synthetic molecules, small molecules and monoclonal antibodies that inhibit MMPs, including MMP9^59^. Among these, small molecule MMP9 inhibitors such as SB-3CT^60^, broad-spectrum MMP inhibitors like Batimastat, Marimastat, Periostat^®^ /Doxycycline, CGS-25,966 and monoclonal antibodies such as REGA-3G12 and DX-2400 are currently in clinical trials^61^. MMP inhibitor-focused preclinical and clinical research has demonstrated potential in reducing tumour progression and metastasis^62^. Strategies for targeting SPARC involve the use of small interfering RNAs (siRNA) to silence SPARC, resulting in an antifibrotic effect^63^, or the use of functional nanoparticles and drugs against cancer^63^. Targeted therapies to inhibit ITGA5 include the potential use of monoclonal antibodies and synthetic RGD (Arginyl-Glycine-Aspartic acid) peptides to block the ITGA5-fibronectin interaction. These approaches may prevent tumour angiogenesis, decrease growth of tumour and metastasis^64^. However, research in this area is still in early stages, targeting MMP9, SPARC and ITGA5 in OSF has the potential to prevent altered ECM remodelling and its contribution to both fibrosis and its progression to cancer.

## Conclusion

A comprehensive methodology involving the integration of bioinformatics, transcriptomic and translational approaches was used for the identification of potential candidate EMT genes. The novel candidate genes SPARC, MMP9, and ITGA5 specifically regulate EMT in OSF and may play a role in malignant transformation. The expression of these genes in OSF and OSFSCC led to the discovery of a novel mechanism that may drive the transition from fibrosis-associated type 2 EMT to invasion-associated type 3 EMT, promoting the malignant transformation of OSF. Functional validation and further investigation can enhance the knowledge on this novel mechanism and aid in the identification of specific therapeutic targets to modulate the progression of fibrosis to malignancy.

## Materials and methods

### Curation of EMT-regulating genes

The list of EMT-regulating genes discovered in pancancer studies was retrieved from the EMTome (http://www.emtome.org/) and dbEMT (http://dbemt.bioinfo-minzhao.org/) databases. A combined list of EMT signatures reported across cancer types was compiled.

### Retrieval of OSF, OSFSCC and OSCC data from databases

To study the expression of these curated EMT signature genes in OSF, OSFSCC (OSF associated with OSCC) and OSCC, relevant datasets were searched in “The Gene Expression Omnibus (GEO) repository” and “The Cancer Genome Atlas Program (TCGA)”. The Gene Expression Omnibus (GEO) repository (https://www.ncbi.nlm.nih.gov/gds) was accessed using the search terms “oral submucous fibrosis OR OSMF OR OSF AND/OR “malignant transformation”. The Cancer Genome Atlas (TCGA) (https://portal.gdc.cancer.gov/) was accessed, and the data pertaining to oral cavity neoplasms, according to the IC10 classification, was retrieved from the following anatomical sites such as lip (C00.9), border of tongue (C02.1), ventral surface of tongue (C02.2), tongue (C02.9), upper gum (C03.0), lower gum (C03.1), gum (C03.9), anterior floor of the mouth (C04.0), floor of mouth (C04.9), cheek mucosa (C06.0), retromolar area (C06.2), and unspecified parts of the mouth (C06.9) was retrieved. The HTSeq-counts data, which is publicly accessible, was retrieved from the Genomic Data Commons (GDC) repository. Patients with insufficient clinicopathologic data were excluded from the study, ensuring that only subjects with complete and reliable information were included in the subsequent analysis. The expression of only the compiled EMT signature genes was analysed in the datasets retrieved from the GEO repository and TCGA-Oral Cavity subset.

### Differential gene expression analysis

The dataset accessed from the GEO repository was analysed for profiling the differentially expressed genes using the interactive web tool GEO2R, an in-built analysis platform. (https://www.ncbi.nlm.nih.gov/geo/geo2r/). Briefly, the false-discovery rate (FDR) was adjusted using Benjamini-Hochberg correction, with a significant cutoff at *p* < 0.05. Limma-voom (Linear models for microarray analysis) pipeline was used to identify the differentially expressed genes (Log2FC > 1, adjusted *p*-value < 0.05) using the microarray datasets in GEO2R platform.

Gene annotation for the HTseq-count dataset derived from the TCGA oral cancer cohort with normal and primary tumours, was performed using the org.HS.eg.db package in the R programming language (v4.1). Low counts (< 10 in more than 20 samples) across the groups were filtered out. Size factors and dispersion estimates for the analysing groups were calculated using the R program. Differential gene expression analysis was performed using DESeq2 R package^65^, with raw HTseq-counts matrices as an input. A log2-fold change of + 1 and − 1 was kept as a threshold with a 5% level of significance and a 1% FDR corrected with Benjamini‒Hochberg correction for the identification of upregulated and downregulated genes.

### Gene set cancer analysis

All the differentially expressed genes (DEGs) retrieved from the GEO dataset and the oral cancer subset of TCGA-HNSC were analysed in GSCALite (http://www.bioinfo.life.hust.edu.cn/web/GSCALite/) to investigate their role in the activation of EMT across different cancers. GSCALite, a web-based platform, serves as a network analysis tool specifically designed for the examination of genomic data in the context of cancer analysis. The platform was utilized to correlate the expression of DEGs with cancer-related pathway activity (activation and inhibition) and with functional states of cancer across 32 cancer types. The top 30 genes activating the EMT pathway across datasets (Supplementary File 1) were curated for further analysis.

### Gene ontology (GO) and pathway enrichment analysis

Gene Ontology (GO) and pathway enrichment analyses were performed for the curated EMT genes using g: Profiler^66^. GO enrichment analysis was used to investigate the molecular functions (MFs), biological processes (BPs), and cellular components (CCs) associated with the curated EMT genes. Pathway enrichment analysis was performed by accessing the KEGG, Wiki Pathways, and Reactome databases using g: Profiler^66^. The hypergeometric test was applied for pathway enrichment analysis, and multiple testing corrections were evaluated using the Benjamini-Hochberg false discovery rate, with a cutoff for the adjusted p-value set at < 0.05.

### Protein‒protein interactions (PPIs)

Cluster analysis was performed for the curated EMT genes using the STRING v10 database (https://string-db.org/), considering a medium confidence score of > 0.4^19^. The integration of various sources, such as high-throughput experimental data, data from databases and the literature, and predictions from genomic context analysis, enabled the identification of protein-to-protein interactions (PPIs)^19^. A cluster analysis was conducted on the curated genes surrounding the nodes of *TGF-β1* and *CDH1* based on our previous in silico work^9^ using the K-means clustering method in STRINGdb.

## Analysis of candidate gene expression through RNA sequencing

The expression of the candidate genes was analysed via whole-transcriptome and mRNA sequencing.

*Whole transcriptome analysis*: RNA sequencing was performed on the NOM (*n* = 2), OSF (*n* = 2) and OSCC (*n* = 2) tissue samples. The isolation of total RNA from the clinical specimens for RNA-seq was performed utilizing the mirVana™ miRNA Isolation Kit (Cat. No. AM1560, Invitrogen, Carlsbad, CA, US) in accordance with the guidelines provided by the manufacturer. Library preparation was conducted utilizing the NEBNext RNA Ultra II kit (Cat. No. E7775, NEB, Massachusetts, US). The libraries that had been prepared were subjected to sequencing using an Illumina HiSeq X Ten/Novaseq/X platform, resulting in the generation of 60 million paired end reads per sample, with each read spanning 150 base pairs. A quality assessment (> Q30) and initial processing of the raw data were performed utilizing the software tools Trimmomatic^67^ and Bowtie2^68^. The pre-processed data was aligned to the human reference genome (hg19) using HiSAT2. Raw read counts were then mapped to Ensemble IDs using feature counts, and subsequently, normalized fragments per kilobase of transcripts per million mapped reads (FPKM) values were computed.

*mRNA sequencing*: To determine the expression of the candidate genes in OSFSCC, matched case-normal total RNA samples (*n* = 2 each) were outsourced for mRNA sequencing (Agilent Technologies, India Pvt. Ltd., India). The paired end (PE) fastq files were processed in the Galaxy Server (https://usegalaxy.org/) (version 23.1.rc1). Briefly, the undesired adapters and low-quality sequences were removed with Cutadapt, and the quality was assessed using MultiQC. Mapping was performed to the human reference assembly (hg19) using RNA STAR to estimate the reads mapped per gene. Furthermore, the fragments per kilobase per million mapped reads (FPKM) estimation per gene was carried out using Cufflinks.

### Validation of the predicted candidate genes by immunohistochemistry

To validate the predicted candidate genes and demonstrate their role in EMT, we performed immunohistochemical analysis on a cohort of 120 clinical tissue specimens consisting of 20 normal (NOM) specimens, 40 OSF specimens, 40 OSCC specimens, and 20 OSFSCC specimens. IHC staining was performed after Institutional Ethics Committee approval was obtained (IED No.: 200/2018), and written informed consent was obtained from all the participants of the study. IHC staining was performed for MMP9 (1:75 dilution, clone: EP127, PathnSitu Biotechnologies, CA, USA), SPARC (1:1000 dilution, clone: ON1-1, Thermo Fisher Scientific, Rockford, USA), and ITGA5 (1:200 dilution, clone: 10F6, Thermo Fisher Scientific, Rockford, USA) according to the manufacturer’s protocols. The IHC Profiler^69^ plugin was used to assess the percentage of cells with positive cytoplasmic staining for MMP9, SPARC and ITGA5. The plugin generates a histogram profile of the DAB image, analysing the pixel intensity and categorizing it as highly positive (+ 3), positive (+ 2), weakly positive (+ 1), or negative (0)^69^. The IHC optical density scores were determined utilizing an established formula, as previously published^70^.

### Statistical analysis

All the statistical analyses were conducted utilizing SPSS (Statistical Package for Social Sciences version 20.0, IBM Corp., NY, USA). One-way analysis of variance (ANOVA) was used to compare the expression across the groups, followed by a post hoc Tukey’s test. A p value < 0.05 was considered to indicate statistical significance. Visualization of the whole-transcriptome sequencing data was performed to assess the expression patterns of the six selected markers using the ggpubr package in the R program (R Core Team (version 2021)). R: A language and environment for statistical computing. Vienna, Austria-based R Foundation for Statistical Computing. https://www.r-project.org).

## Electronic supplementary material

Below is the link to the electronic supplementary material.

Supplementary Material 1

## Data Availability

The datasets generated and analysed during the current study is available in the Gene Expression Omnibus, mRNA-sequencing raw data in GSE274202 and RNA sequencing raw data in GSE274203, Weblink: https://www.ncbi.nlm.nih.gov/geo/query/acc.cgi? accTemporary GSE Data Code: gxmtkccehtsxdyx (GSE274202), ofsfimmeppaxjct (GSE274203).
