# Supplementary material for: Novel transcripts of EMT driving the malignant transformation of oral submucous fibrosis
Source: Sci Rep. 2025 Jan 26;15:3294. doi: 10.1038/s41598-025-87790-2 (PMC11770107; doi:10.1038/s41598-025-87790-2)
Supplement: Supplementary file 1 — Supplementary Material 1 [file 41598_2025_87790_MOESM1_ESM.pdf]

Supplementary Figures

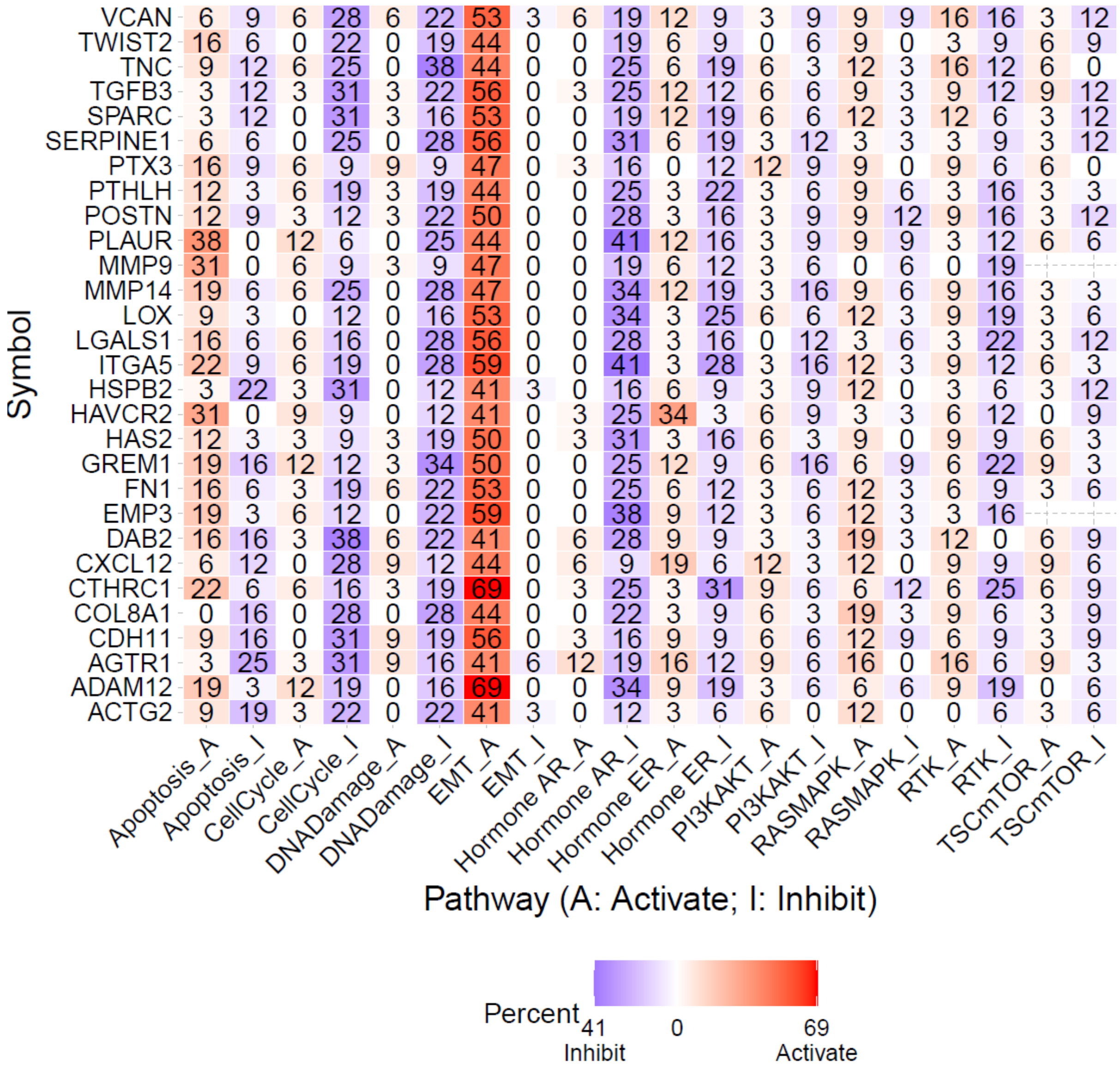

Supplementary Figure 1. The heat plot shows the pathway activity curated by the EMT gene list, showing the activation of EMT. The number indicates the percentage of cancers in which the genes had an activation effect.

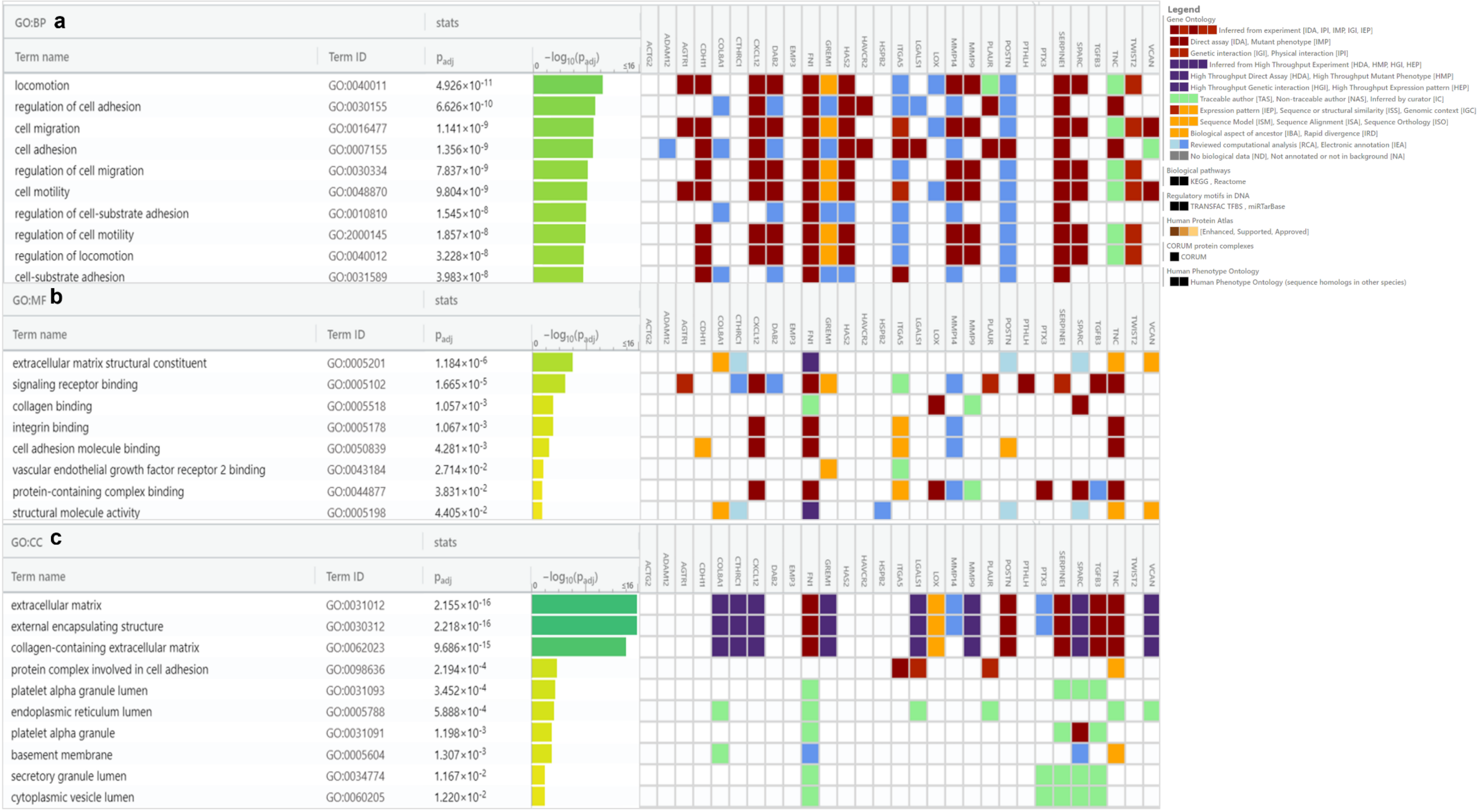

Supplementary Figure 2. Gene Ontology for the curated EMT gene list showing enriched biological processes **(a),** molecular functions **(b)** and cellular components **(c).**

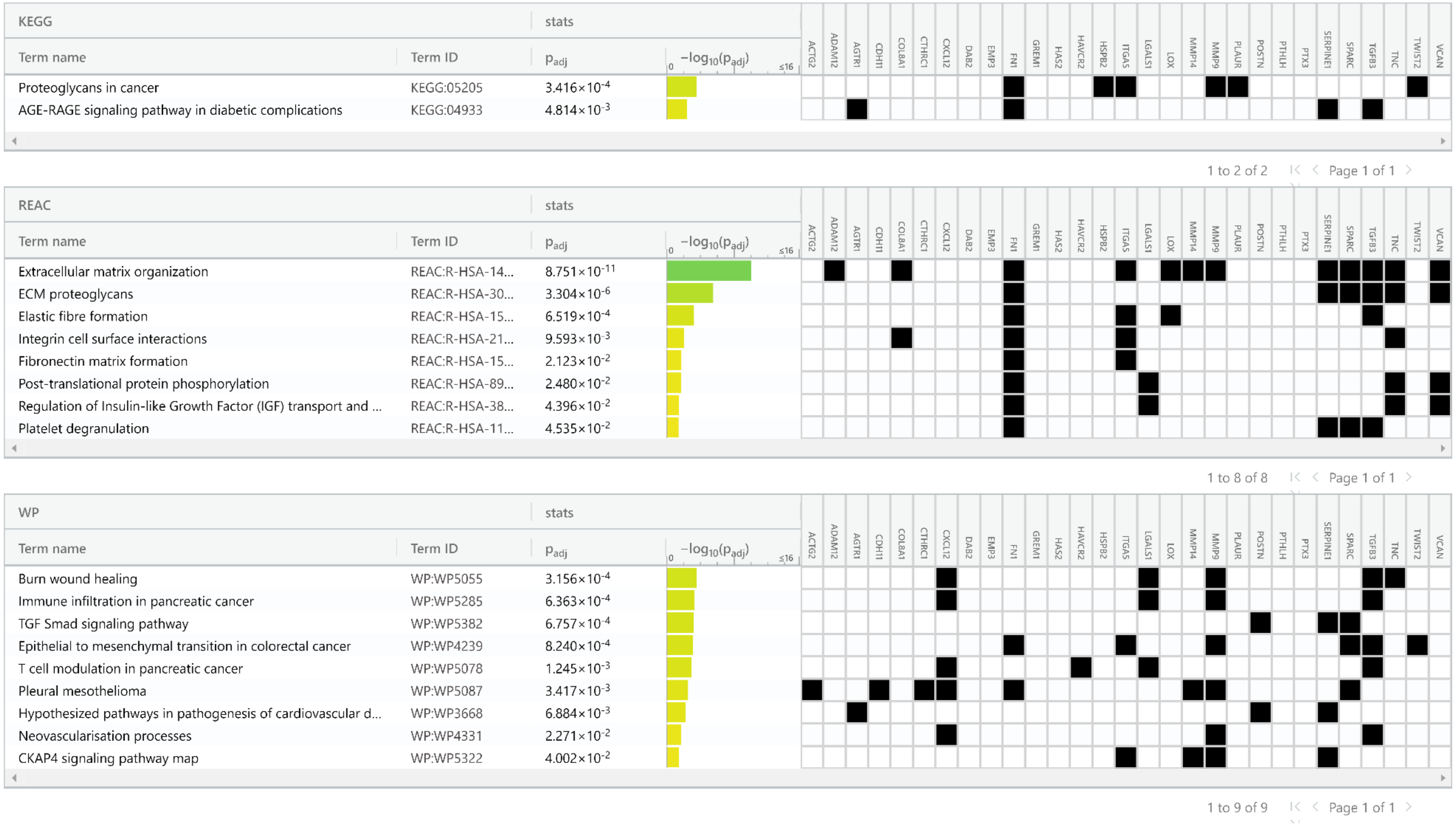

Supplementary Figure 3. Gene Ontology for the curated EMT gene list showing enriched pathways in the KEGG, Reactome and Wiki Pathways databases.
